# Comparison of ^68^Ga-PSMA PET/CT with fluoride PET/CT for detection of bone metastatic disease in prostate cancer

**DOI:** 10.1186/s41824-022-00127-4

**Published:** 2022-03-01

**Authors:** Naresh Regula, Vasileios Kostaras, Silvia Johansson, Carlos Trampal, Elin Lindström, Mark Lubberink, Victor Iyer, Irina Velikyan, Jens Sörensen

**Affiliations:** 1 Division of Radiology and Nuclear Medicine, Department of Surgical Sciences, Uppsala University, Uppsala, Sweden; 2 Divison of Oncology, Department of Immunology, Genetics and Pathology, Uppsala University Hospital, Uppsala, Sweden; 3 Department of Medical Physics, Uppsala University Hospital, Uppsala, Sweden; 4 Department of Medical Imaging, Uppsala University Hospital, Uppsala, Sweden

**Keywords:** PSMA, Fluoride, Prostate cancer, PET/CT

## Abstract

**Background:**

^18^F-NaF positron emission tomography/computed tomography (fluoride PET/CT) is considered the most sensitive technique to detect bone metastasis in prostate cancer (PCa). ^68^Ga-PSMA-11 (PSMA) PET/CT is increasingly used for staging of PCa. This study primarily aimed to compare the diagnostic performance of fluoride PET/CT and gallium-based PSMA PET/CT in identifying bone metastasis followed by a comparison of PSMA PET/CT with contrast-enhanced CT (CE-CT) in identifying soft tissue lesions as a secondary objective.

**Methods:**

Twenty-eight PCa patients with high suspicion of disseminated disease following curative treatment were prospectively evaluated. PET/CT examinations using fluoride and PSMA were performed. All suspicious bone lesions were counted, and the tracer uptake was measured as standardized uptake values (SUV) for both tracers. In patients with multiple findings, ten bone lesions with highest SUV_max_ were selected from which identical lesions from both scans were considered for direct comparison of SUV_max_. Soft tissue findings of local and lymph node lesions from CE-CT were compared with PSMA PET/CT.

**Results:**

Both scans were negative for bone lesions in 7 patients (25%). Of 699 lesions consistent with skeletal metastasis in 21 patients on fluoride PET/CT, PSMA PET/CT identified 579 lesions (83%). In 69 identical bone lesions fluoride PET/CT showed significantly higher uptake (mean SUV_max_: 73.1 ± 36.8) compared to PSMA PET/CT (34.5 ± 31.4; *p* < 0.001). Compared to CE-CT, PSMA PET/CT showed better diagnostic performance in locating local (96% vs 61%, *p* = 0.004) and lymph node (94% vs 46%, *p* < 0.001) metastasis.

**Conclusion:**

In this prospective comparative study, PSMA PET/CT detected the majority of bone lesions that were positive on fluoride PET/CT. Further, this study indicates better diagnostic performance of PSMA PET/CT to locate soft tissue lesions compared to CE-CT.

**Supplementary Information:**

The online version contains supplementary material available at 10.1186/s41824-022-00127-4.

## Background

In prostate cancer (PCa) patients, spread to the skeletal system is common with progressive disease and approximately 80% of patients show bone metastasis in advanced stages (Herrera and Berthold 2015; Hernandez et al. 2018). Bone provides an environment rich in factors that facilitate survival and stimulate growth of metastatic tumour cells. Interaction of tumour cells with local bone matrix leads to an osseous response dominated by excess osteoblastic activity (Fornetti et al. 2018; Buenrostro et al. 2016; Wang et al. 2020; Quiroz-Munoz et al. 2019), resulting in formation of predominantly osteoblastic bone lesions most commonly located in axial skeleton.

The presence of bone metastasis has a profound impact on patient prognosis with a shorter cancer specific mortality-free survival of 24 months (Gandaglia et al. 2015). Thus, accurate early detection of bone spread throughout PCa disease progression is important to reduce potential complications and to provide optimal treatment. This challenge is further amplified by the continuous development of new imaging techniques. Through a range of imaging techniques currently available to detect bone lesions, the choice of selection for the clinicians is complex. Further, the chosen imaging modality must be able to accurately visualize the site of bone metastasis.

Bone scintigraphy (BS) with [^99m^Tc]Tc-methylene diphosphonate (MDP) is used in the diagnostic workup of PCa patients with bone lesions. BS is a relatively inexpensive technique with the advantage of broad availability and a large body of validation. On BS, MDP is incorporated into the hydroxyapatite matrix of bone in proportion to osteoblastic activity and allows the visualization of bone lesions. The tumour volume on a BS can be quantified with bone scan index (BSI) (Ulmert et al. 2012; Nakajima et al. 2017), an independent prognostic biomarker of survival (Ali et al. 2020; Wiyanto et al. 2017; Armstrong et al. 2018; Miyoshi et al. 2017). Currently, conventional BS is still considered the international standard and recommended in the guidelines for management of PCa patients with bone metastasis (Cornford et al. 2017; Mottet et al. 2017). However, BS has several limitations such as poor anatomical correlation, low sensitivity and specificity. As per European Association of Urology (EAU) guidelines BS should not be recommended in biochemical relapse patients with PSA below 10 ng/mL due to high probability for negative findings (Cornford et al. 2017; Mottet et al. 2017). Further, BS is often complemented by diagnostic grade contrast-enhanced CT (CE-CT) to rule out false positive uptake in focal degenerative bone disease, evaluate fracture risk and diagnose soft tissue metastasis.

Prior to use of [^99m^Tc]Tc-MDP, [^18^F]-sodium fluoride (NaF) with a similar uptake mechanism was approved for bone imaging but the relatively short half-life and high energy of ^18^F limited its use with gamma cameras at that time. However, the growing popularity of PET/CT with improved detection prompted the resurgence of fluoride PET/CT. Further, more rapid blood clearance, high bone-to-background ratio and shorter examination time favoured the use of fluoride PET/CT over BS. Several studies have shown superiority of fluoride PET/CT compared to BS in terms of sensitivity and specificity (Beheshti et al. 2016; Langsteger et al. 2016; Cook et al. 2016).

With respect to other PET tracers, accuracy of ^18^F- or ^11^C-choline PET/CT in detection of bone lesions was identical but with higher specificity compared to fluoride PET/CT (Beheshti et al. 2016; Wondergem et al. 2013). Further, choline PET/CT showed promising results for the early detection of bone metastasis (Beheshti et al. 2016). A systematic review from 2016 concluded that fluoride-based, acetate-based and choline-based PET/CT are the most sensitive and adequate imaging techniques (Wibmer et al. 2016). In recent years, ^68^Ga-PSMA-11, targeting prostate-specific membrane antigen (PSMA), was successfully introduced into clinical practice (Afshar-Oromieh et al. 2015, 2013). Results from comparative studies showed that PSMA PET/CT outperformed commonly used tracers in localizing lesions in PCa recurrent patients (Diao Wei and Lio 2019; Calais et al. 2019; Afshar-Oromieh et al. 2014).

At our institution fluoride PET/CT is commonly recommended over BS to identify bone metastasis in PCa patients at primary staging of high-risk cancer, at biochemical relapse. PSMA PET/CT was recently introduced at our hospital with the intention to use PSMA PET/CT as the primary tool for restaging of PCa. To enable PSMA PET/CT for clinical routine, both scans need to be compared and validated. Therefore, the primary aim of this study was to compare and evaluate diagnostic performance of fluoride PET/CT and PSMA PET/CT in identifying bone metastasis in PCa relapse patients. Further, comparison of PSMA PET/CT and CE-CT to detect soft tissue lesions was set as a secondary objective.

## Methods

### Patient characteristics

Twenty-eight patients were internally referred for PET imaging with high suspicion of widespread disease. The inclusion criteria for this prospective study were: histologically confirmed adenocarcinoma of the prostate, primary treatment followed by secondary and/or third-line therapy, and rising PSA levels with high likelihood of suspicious cancer spread prior to the PET examinations. Three patients had curative first-line therapy, five patients were given second-line treatment following first-line therapy and in 20 patients additional third-line therapy was offered prior to PET scan. In this prospective study both PSMA and fluoride PET/CT scans were acquired within 1 week in 27 subjects and in one subject the time interval was 15 days. All relevant clinical data including PSA at time of scan, PSA doubling time (PSA_DT_), PSA velocity (PSA_Vel_), Gleason score (GS) and age were recorded. The study was approved by the regional ethics review board (Dnr. 2017/190). Written informed consent was obtained from all research subjects.

### Production of ^68^Ga-PSMA-11 and ^18^F-NaF

^68^Ga-PSMA-11 (^68^Ga-PSMA-11: ^68^Ga-Glu-NH-CO-NH-Lys(Ahx)-HBED-CC (ABX, Germany) was synthesized on a fully automated synthesis platform (Modular-lab, PharmTracer; Eckert & Ziegler, Eurotope, Berlin, Germany) using dedicated disposable cassettes (C4-Ga68-PSMA, Eckert & Ziegler, Germany) (Eder et al. 2012) in accordance with good manufacturing practice (GMP) guidelines. Pharmaceutical grade ^68^Ge/^68^Ga generator (GalliaPharm, 50 mCi, Eckert&Ziegler, Germany) was used to produce gallium-68. The product was formulated in saline containing less than 10% of ethanol and sterile filtered (0.22 µm).

^18^F-NaF was produced on in-house built automated system on a cyclotron (17 MeV, Scanditronix) from H_2_^18^O and was trapped on the QMA-cartridge that then was washed with sterile water. The product was eluted with sterile physiological phosphate buffer saline and passed through a sterile 0.22 µm filter.

### PET/CT imaging protocol

All PET/CT examinations were performed on a Discovery MI PET/CT system (GE Healthcare, Waukesha, WI) with a spatial resolution of 4 mm at the centre of the field of view and 2 min acquisition per bed position. After obtaining a CT transmission scan (140 kV, 40–80 mA) without contrast medium, emission scans from mid-thigh to skull base were acquired. PET scans were acquired 63 ± 5 min (range 59–75 min) after intravenous administration of 1.6 ± 0.5 MBq/kg (range 0.8–2.7 MBq/kg) of PSMA and 64 ± 12 min (range 47–97 min) after intravenous administration of 3.1 ± 0.3 MBq/kg (range 2.4–3.8 MBq/kg) of fluoride. A CE-CT scan was performed immediately after fluoride PET.

PSMA PET images were reconstructed using a block-sequential regularized expectation maximization (BSREM) (Q.Clear; GE Healthcare) method with β-value 900 (Lindström et al. 2019). Ordered subsets expectation maximization (OSEM) (VPFX-S; GE Healthcare) method with 3 iterations, 16 subsets and a 5-mm Gaussian post-processing filter was used for Fluoride PET image reconstruction.

### Image analysis

Hermes Hybrid Viewer version 2.0.0 (Hermes Medical Solutions AB, Stockholm, Sweden) was used for PET/CT image analysis. All bone lesions with focal uptake above the background activity of normal bone and high suspicion for malignancy were identified on both PSMA and fluoride PET/CT scans. Degenerative bone lesions, bone fractures and other lesions with non-specific uptake were ruled out and excluded based on findings from CE-CT and inter-observer agreement. Along with bone metastases, soft tissue lesions were also counted on PSMA PET/CT. Two observers (V.I. and N.R.) independently reviewed CE-CT findings for identification of metastatic bone lesions, and one of the observers (V.I.) was completely blinded to PET information.

Tracer uptake in positive lesions was measured as standardized uptake values (SUV). SUV was defined as a ratio of radioactivity concentration in region of interest (Bq/mL) and injected dose (Bq) divided by body weight (g). Maximum and mean SUV (SUV_max_, SUV_mean_) and tumour volume (TV) were calculated by placing a volume of interest (VOI) over pathological lesions having a fixed isocontour threshold (Fluoride: threshold of 50% SUV_max_; PSMA: threshold of 40% SUV_max_) for all lesions. Total tumour volume (TTV = sum of TV of all lesions) and total lesion activity (TLA = sum of SUV_max_*TV of all lesions) were calculated for all positive bone lesions on fluoride and PSMA PET/CT. Due to higher reproducibility, SUV_max_ was chosen for comparison. In patients having multiple bone lesions, ten bone lesions with highest SUV_max_ were selected from which identical bone lesions on both PET scans were considered for direct comparison of SUV_max_ among the two scans.

### Statistical analysis

Data were presented as mean ± SD. Wilcoxon signed-rank test was used for comparison of SUV_max_ of bone lesions. McNemar test was used to compare the number of lesions on PSMA and fluoride PET/CT scans. Inter reader agreement on reviewing CT findings was evaluated using kappa coefficient. SUV_max_ from both PET scans were normalized using log transformation and the correlation among them was examined using univariate analysis.

## Results

Patient characteristics are summarized in Table 1. Average age of the patients was 70.4 ± 7.4 years (range 55–82, median 70) with a mean PSA level of 205 ± 688 ng/mL (range 2.2–3456, median 23) at time of diagnosis. Mean PSA measured at time of the first scan was 51 ± 93 ng/mL (range 0.7–341, median 7.6).

**Table 1:** Patient characteristics. Gleason grade group (GG) was defined using revised International Society of Urological Pathologists (ISUP) system. Both GG and PSA at diagnosis were not retrievable in two subjects

Patient no.	Age (years)	ISUP GG	PSA at diagnosis	PSA at scan	Fluoride (MBq)	PSMA (MBq)	Time diff. (days)
1	62	2	2.2	29	264	182	7
2	65	3	71	4.4	256	142	7
3	78	–	38	0.7	194	175	5
4	62	1	8.7	9.5	337	196	5
5	82	–	3456	276	231	150	2
6	80	4	200	1.9	227	163	2
7	73	1	9	341	258	144	2
8	73	5	6.9	2	307	164	7
9	78	5	26	8	239	149	7
10	75	3	7.1	42	260	123	7
11	69	4	7.2	4	256	162	7
12	76	3	7.1	5.8	256	152	7
13	64	5	112	171	268	123	7
14	65	5	9	2.3	257	186	15
15	62	5	28	32	322	183	7
16	67	5	30	20	293	114	7
17	76	5	950	7.2	333	196	7
18	79	1	18	211	292	132	7
19	65	4	69	25	263	102	7
20	80	2	9	4.3	230	91	7
21	82	2	80	177	299	138	7
22	66	3	–	6.7	295	175	7
23	69	2	–	4	309	121	7
24	61	2	128	19	280	101	7
25	55	3	5.4	4.7	422	120	7
26	74	1	20	9.4	269	115	7
27	70	3	27	2.7	246	86	5
28	64	3	16	1.9	300	83	5

No adverse reactions were observed in any of the patients after administration of fluoride and PSMA. Both scans were negative for bone metastases in 7 patients (25%). However, PSMA PET/CT detected at least one positive finding of local and/or lymph node metastases in all 7 subjects. Mean PSA at time of scan in patients with positive bone lesions on PET scans (64.4 ± 103.5 ng/mL) was significantly elevated, compared to patients with negative bone findings (10.0 ± 14.3 ng/mL; *p* = 0.03).

**Fig. 1:**
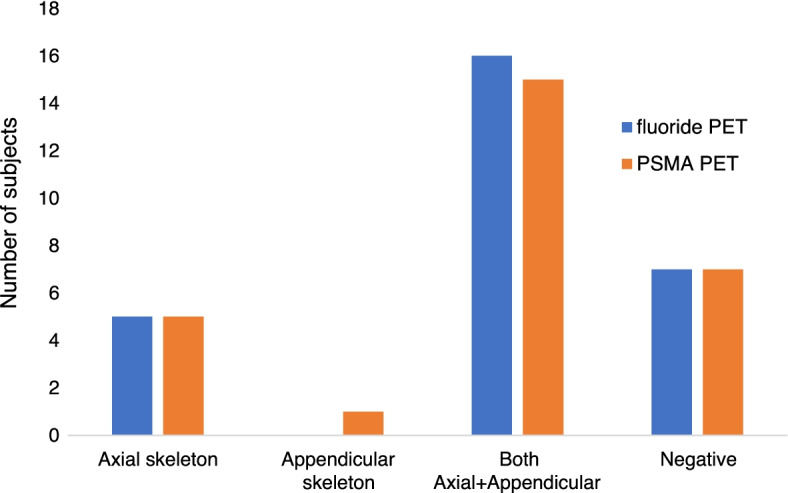
Pattern of detected bone lesions on both fluoride and PSMA PET. Seven patients showed negative findings and five subjects showed positive bone metastases in the axial skeleton on both scans. Bone lesions with axial and appendicular skeleton were detected in 16 patients on fluoride PET and 15 subjects on PSMA PET. In one patient bone lesions in appendicular skeleton were detected only on PSMA PET

Bone lesions were categorized into axial (spine, ribs, sternum and skull) and appendicular (upper and lower limbs and pelvis) skeletal lesions. The pattern of detected bone lesions is shown in Fig. 1. Both scans identified bone metastases limited to axial skeleton in five patients (18%). Patients with both axial and appendicular skeletal lesions on fluoride and PSMA PET/CT scans were 16 (57%) and 15 (54%), respectively. Positive bone lesions in appendicular skeleton alone were seen with PSMA PET/CT in one subject.

**Fig. 2:**
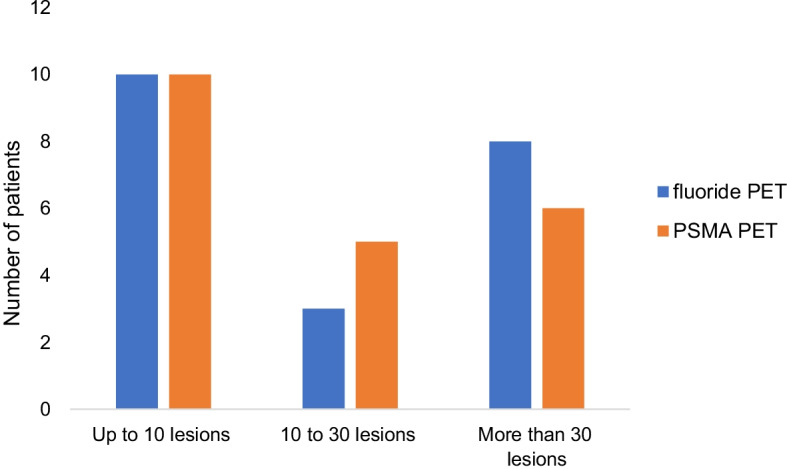
Patients were stratified based on number of bone lesions detected on fluoride and PSMA PET, excluding the negative PET scans. Both PET scans showed up to 10 bone lesions in 10 subjects. Fluoride PET showed 10–30 bone lesions in three patients whereas PSMA identified 10–30 lesions in five subjects. Multiple bone lesions (more than 30) were detected in eight patients on fluoride PET. PSMA PET identified multiple bone lesions in six patients

In 21 of 28 included patients, 699 lesions consistent with bone lesions were detected with fluoride PET/CT. In contrast, PSMA PET/CT identified 579 bone lesions (83% of positive fluoride PET/CT lesions, *p* < 0.001) considered positive for bone metastasis. All bone lesions detected on PSMA PET/CT were also seen on fluoride PET/CT. Stratification of the patients based on number of lesions is shown in Fig. 2. Fluoride PET/CT showed less than 10 lesions in 10 patients, up to 30 lesions in three subjects and more than 30 lesions in eight patients. Whereas, PSMA PET/CT detected less than 10 lesions in 10 patients, up to 30 lesions in five subjects and more than 30 lesions in six patients. Bone metastases up to five lesions were considered as oligometastatic bone disease, fluoride PET/CT identified this in six patients whereas nine patients were detected on PSMA PET/CT. Along with bone lesions, PSMA PET/CT identified local relapse in the prostatic fossa in 7 patients and 36 positive lymph node lesions in 9 patients. Four subjects showed both positive local relapse and lymph node lesions (n = 15).

Sixty-nine identical bone lesions in 21 patients with tracer uptake on both scans were included for a quantitative comparison. Mean SUV_max_ was significantly higher on fluoride PET/CT compared to PSMA PET/CT (73 ± 37 vs 35 ± 31; *p* < 0.001). TTV from bone lesions on PSMA PET/CT strongly correlated with fluoride PET/CT TTV (r = 0.9, *p* < 0.001).

Reviewing of CE-CT scans showed widespread disease (> 30 lesions) in three patients and were excluded. In the remaining 25 patients, the total number of bone lesions detected by the observers in CE-CT were 120 (V.I.) and 136 (N.R.), respectively. Substantial agreement was observed between the readers (89%, Cohen’s k = 0.72) in locating bone lesions.

**Fig. 3:**
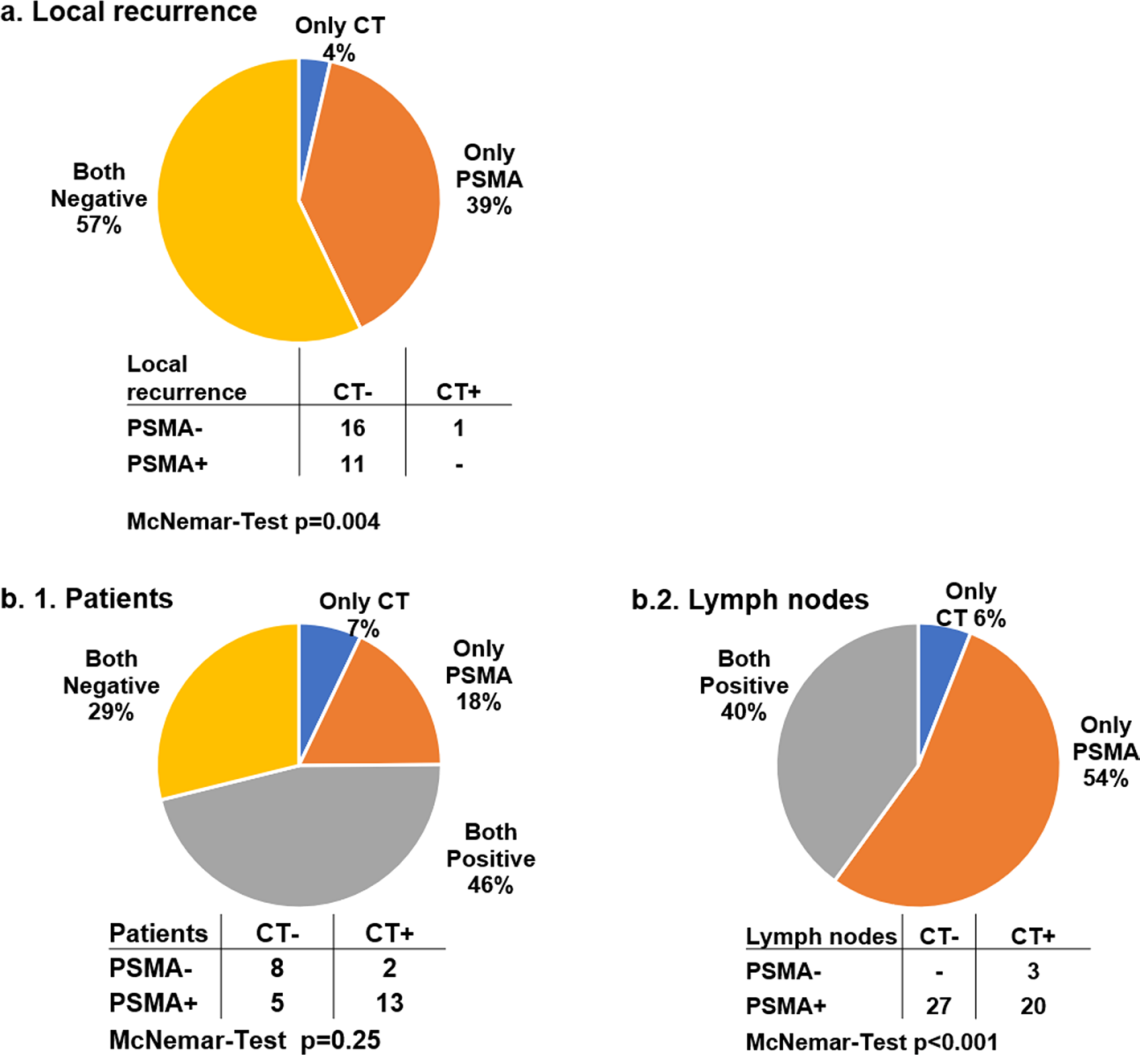
Suspicious local and lymph node lesions in patients with biochemical relapse. **a** Percentage and numbers of local recurrence in the prostatic fossa detected by CT and PSMA PET. **b.1** Percentage and number of patients with suspicious lymph nodes detected by CT and PSMA PET. **b.2** Percentage and number of suspicious lymph nodes identified by diagnostic CT and PSMA PET

Findings from one blinded observer (V.I.) were considered for comparison of soft tissue lesions with PSMA PET/CT. In 12 of 28 patients, local relapse in the prostatic fossa was found, of which 11 lesions were detected with PSMA PET/CT, whereas CE-CT showed only one lesion (Fig. 3). A total of 50 lymph node lesions suspicious for cancerous lesions in 20 patients were detected using CE-CT or PSMA PET/CT (Fig. 3). Twenty-seven of 50 lymph nodes were only seen on PSMA PET/CT, whereas CE-CT alone was positive for 3 lymph nodes. Both CE-CT and PSMA PET/CT detected 20 lymph node lesions in 13 patients.

All patients had clinical follow-up and additional imaging scans at different time points were available in 21 subjects (CT in 9, fluoride PET/CT in 4, PSMA PET/CT in 4, BS in 2, WB-MRI in one and ultrasound in one subjects). Follow-up scans were not available in seven patients. Among the four subjects with PSMA PET/CT at follow-up several bone lesions with initially fluoride + /PSMA- findings were PSMA-positive at follow-up. A few instances of PSMA + /fluoride- lesions were seen in subjects with widespread bone disease, but none of these lesions could be adequately evaluated by follow-up imaging.

## Discussion

This was a prospective study in PCa patients with suspected bone metastases to evaluate the performance of PSMA PET/CT compared to fluoride PET/CT. The results suggested that PSMA PET/CT was able to detect most of the bone lesions (83%) that were positive on fluoride PET/CT.

The usefulness of PSMA PET/CT has primarily been investigated with a focus on localizing biochemical relapse of PCa (Afshar-Oromieh et al. 2015, 2014; Eiber et al. 2015). A number of studies (summarized in Additional file 1: Table S1) have investigated the diagnostic accuracy of PSMA PET/CT regarding bone metastasis compared to BS (Pyka et al. 2016; Janssen et al. 2018). In addition, several studies compared the diagnostic performance of several clinically available imaging modalities in localizing bone spread in PCa patients (Dyrberg et al. 2019; Fonager et al. 2017; Jambor et al. 2016; Lengana et al. 2018; Poulsen et al. 2014; Zacho et al. 2018a, b; Zacho et al. 2020; Madsen et al. 2020; Harmon et al. 2018). These studies showed better diagnostic performance of fluoride PET/CT and PSMA PET/CT compared to other modalities such as BS, SPECT/CT, WB-MRI and choline PET/CT (see Additional file 1: Table S1 for details). Further, PSMA PET/CT showed additional value with improved specificity but with an overlapping sensitivity in comparison to fluoride PET/CT. The results from this study on comparison of PSMA PET/CT with fluoride PET/CT were also in line with these studies.

**Fig. 4:**
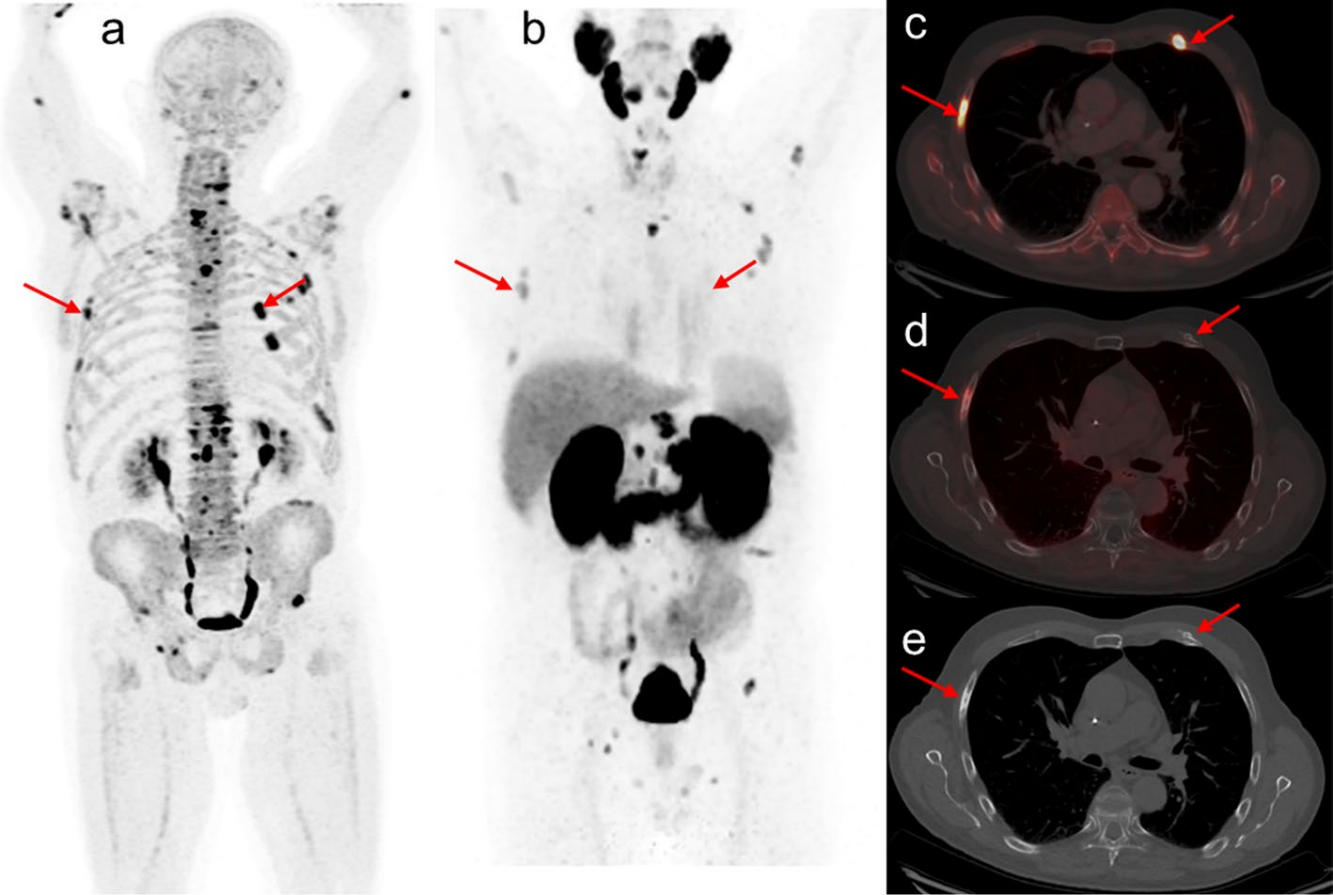
A 65-year-old patient diagnosed with 4 + 3 PCa underwent radiation, hormonal and chemotherapy and became hormone refractive. On referral, PET imaging revealed bone lesions on maximum intensity projection of fluoride PET (**a**) and PSMA PET (**b**). The majority of bone lesions were sclerotic in nature having high intensity uptake on fluoride PET (**a**) but reduced uptake on PSMA PET (**b**). Fused transaxial PET/CT images showing two sclerotic rib lesions on fluoride PET (**c**), whereas PSMA PET showed only one sclerotic lesion (d, left arrow over rib). Respective sclerotic lesions showed on axial CT (**e**)

**Fig. 5:**
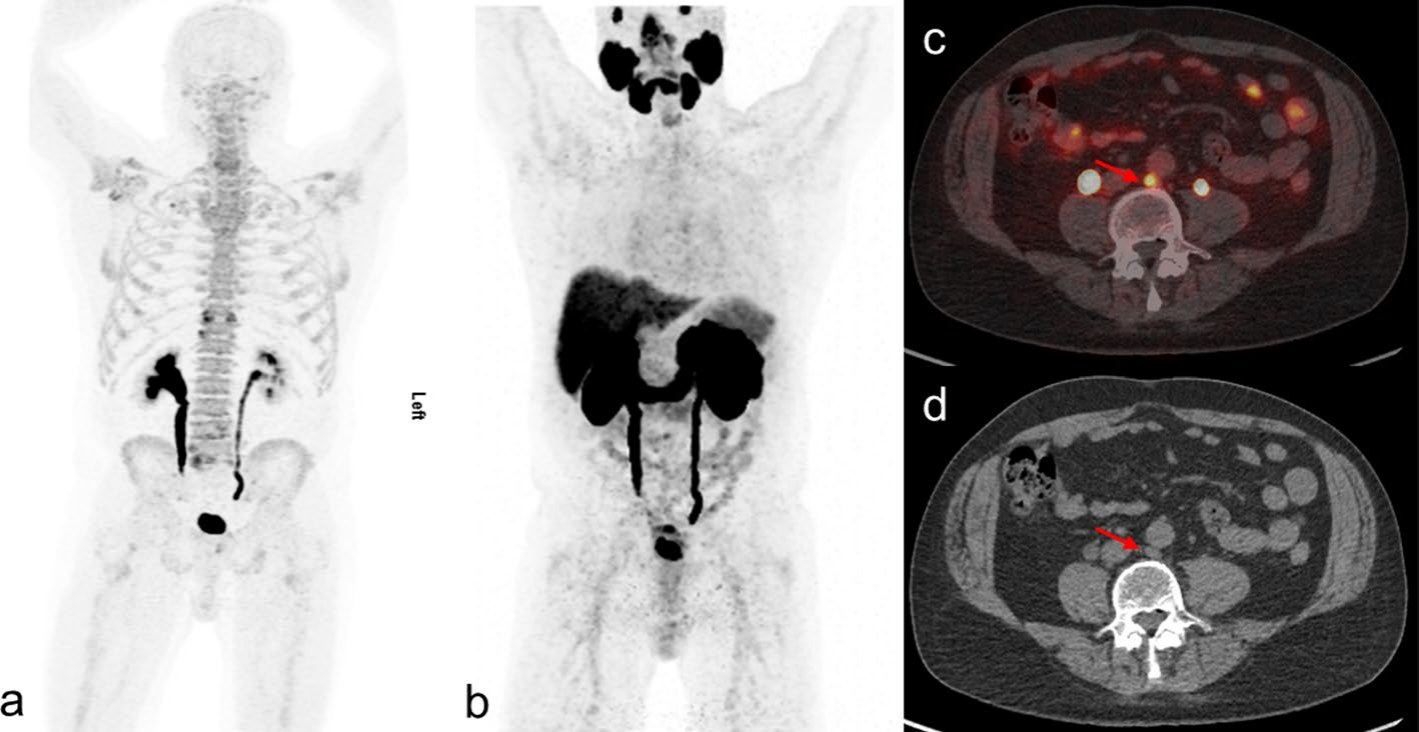
A 75-year-old 4 + 3 PCa patient treated with radiation and adjuvant hormonal therapy referred for PET imaging due to rising PSA (PSA was 42 ng/mL at time of scan). Maximum intensity projection images of both fluoride PET (**a**) and PSMA PET (**b**) showed negative bone lesions. However, para-aortic lymph node (red arrow) showed positive uptake on PSMA PET (**c**), which also correlated with CT (**d**)

One important finding from this study is a higher detection rate of fluoride PET/CT compared to PSMA PET/CT (699 vs 579 bone lesions). In comparison to our study, a retrospective study with a smaller dataset (n = 16) conducted by Uprimny et al. also documented higher detection rate of fluoride PET/CT (Uprimny et al. 2018). In that study, the authors observed low uptake of PSMA in osteosclerotic lesions similar to Eiber et al. (2015), stated as the possible explanation for low detection rate of bone lesions on PSMA PET/CT. In concordance, we also noticed overall low intensity of PSMA uptake in sclerotic bone lesions (Fig. 4). However, PSMA PET had an influential role with its ability to detect soft tissue PCa spread. In seven patients without bone disease on both PET scans, the presence of lymph node lesions on PSMA PET/CT changed the treatment decision (Fig. 5). We used ^68^Ga-PSMA-11 in this study, but the superior sensitivity of ^18^F-fluoride for bone lesions has been documented for other PSMA-based radiopharmaceuticals (Fourquet et al. 2021). Findings from a comparative study using all comparative imaging data for PET radiotracers in recurrent PCa confirmed the superiority of the three most commonly used PSMA radiotracers (^68^Ga-PSMA-11, ^18^F-PSMA-1007 and ^18^F-DCFPyL) with a large overlap between ^68^Ga and ^18^F-labelled PSMA-radiotracers with regard to patient-level detection rates (Alberts et al. 2021a). Further, a direct comparison between ^68^Ga-PSMA-11, ^18^F-PSMA-1007 in terms of clinical performance and cost efficacy showed non-significantly higher PET positivity rate but significantly greater rates of uncertain findings for ^18^F-PSMA-1007, whereas as cost efficacy analysis based on available health economic data favoured ^68^Ga-PSMA-11 in majority jurisdictions (three of four) (Alberts et al. 2021b). Though logistical and clinical advantages of low urinary excretion seems to favour the use of ^18^F-fluoride-based radiotracers over ^68^Ga-based PSMA-tracers, further studies are needed to advocate this with certain.

Determining the presence of oligometastatic bone lesions, the potential targets for metastatic-directed therapies are clinically relevant as they can be irradiated. In the six patients who were identified with oligometastatic bone lesions with both PSMA and fluoride PET/CT, one patient showed additional non-osseous lesions on PSMA PET/CT, altering the treatment plan. The remaining five subjects received radiation therapy. Three additional patients were identified with oligometastatic bone status on PSMA PET/CT but had more than five lesions on fluoride PET/CT. In addition to bone lesions, PSMA PET/CT also showed either local relapse or lymph node lesions in these patients which influenced the treatment management. However, this cohort is not large enough to determine the added value of PSMA PET/CT treatment decisions related to oligometastatic disease.

The choice of imaging techniques for restaging of PCa at a given centre depends on local parameters such as cost-effectiveness, accessibility and expertise. Many hospitals around the world still use BS along with an abdominal CE-CT scan as standard-of-care in the diagnostic workup. Although CE-CT has limited sensitivity (Yang et al. 2011), findings particularly of skeletal lesions from CE-CT together with bone scan findings has clinical implication in many hospitals where access to PET/CT imaging is limited. Hence, comparison of CE-CT findings with PSMA PET/CT is relevant to confirm the better diagnostic performance of the latter. Fluoride PET/CT is generally considered superior to ^99m^Tc-MDP-BS and ^99m^Tc-MDP-SPECT/CT for detection of bone metastasis (Poulsen et al. 2014; Bortot et al. 2012; Iagaru et al. 2012). In further support, few studies evaluated the impact of fluoride PET/CT on patient prognosis (Hillner et al. 2018; Gareen et al. 2018). At our hospital, fluoride PET/CT has been recommended over BS in PCa restaging to locate early signs of bone disease and is generally performed with CE-CT to detect soft tissue lesions.

Using follow-up scanning we could show that some lesions are detected earlier with fluoride PET/CT than with PSMA PET/CT. In none of these cases did the higher sensitivity of fluoride PET/CT lead to therapy changes and the measured tumour burden was similar for both tracers. However, equivocal skeletal findings on fluoride PET/CT are relatively common and contribute to false positive cases (Fonager et al. 2017; Poulsen et al. 2014), which might require additional diagnostic procedures. In this study, we also found equivocal bones lesions on PSMA PET/CT, but the overall perception is that this is a lesser problem with PSMA than with fluoride PET/CT. In addition, PSMA PET/CT provided additional information in detecting local recurrences and lymph node metastases, thus influencing the management, as seen in 7 subjects in our current study.

Based on this study and the vast literature already published, ^68^Ga-PSMA PET/CT is a highly relevant first-line imaging modality in recurrent PCa. However, fluoride PET/CT had a significantly higher detection rate for bone metastases and could be a relevant complement to PSMA PET in certain scenarios. PSMA-expression might be reduced or absent in dedifferentiated PCa and a discrepancy between PSA levels and PSMA PET findings should potentially trigger additional imaging with higher sensitivity in biochemical relapses after treatments with curative intent (McGeorge et al. 2021). PSMA PET/CT followed by fluoride PET/CE-CT might also be a relevant combination for ruling out PSMA-negative lesions before treatment with PSMA-targeted radionuclide therapies.

This study has several limitations. It was based on a small group of patients with high suspicion for widespread disease involving bone, introducing some bias. A standard reference, preferably histological reports, to confirm the discrepant findings of PET imaging is missing. However, accessing bone for biopsy collection is neither ethically nor practically possible in all lesions. Follow-up scans with optimal imaging modalities were not available in all patients.

## Conclusions

In this prospective comparative study, fluoride PET/CT identified significantly more bone lesions compared to PSMA PET/CT. However, better diagnostic performance of PSMA PET/CT to locate soft tissue lesions compared to CE-CT favours the use of PSMA PET/CT as the more relevant molecular imaging method for restaging of PCa recurrence.

## Supplementary Information

**Additional file 1**. Table S1.

## Data Availability

The datasets used and/or analysed during this study are available from the corresponding author on reasonable request.
