# Supplementary material for: Comparison of 68Ga-PSMA PET/CT with fluoride PET/CT for detection of bone metastatic disease in prostate cancer
Source: Eur J Hybrid Imaging. 2022 Mar 1;6:5. doi: 10.1186/s41824-022-00127-4 (PMC8885936; doi:10.1186/s41824-022-00127-4)
Supplement: Supplementary file 1 — Additional file 1. Table S1. [file 41824_2022_127_MOESM1_ESM.docx]

Supplementary Table 1. Studies comparing the diagnostic performance of different imaging techniques to locate bone metastases in prostate cancer patients.

| Authors | Study type (year) | Number of subjects | PSMA tracer | Other technique | Other technique | Inclusion criteria | Mean age at PET (age) | Median PSA at PET ng/mL | Patient characteristics |
| --- | --- | --- | --- | --- | --- | --- | --- | --- | --- |
| Dyrberg et.al [1] | Prospective (2018) | 55 | ^68^Ga-PSMA-11 | ^18^F-NaF PET/CT | WB-MRI | Confirmed prostate cancer patients referred for initial staging (n=10) and for follow-up (n=45) | 75 y | NR | Patient based diagnostic performance (sensitivity, specificity, accuracy):  PSMA PET/CT- 100%, 100%, 100%  NaF PET/CT- 95%, 97%, 96%  WB-MRI- 80%, 83%, 82% |
| Fonager et.al [2] | Prospective (2017) | 37 |  | ^18^F-NaF PET/CT | BS, SPECT/CT | Newly diagnosed, high risk prostate cancer | 71 y | NR | Patient based diagnostic performance (sensitivity, specificity, PPV, NPV):  NaF PET/CT- 89%, 90%, 96%, 75%  SPECT/CT- 89%, 100%, 100%, 77%  BS- 78%, 90%, 96%, 60% |
| Jambor et.al [3] | Prospective (2015) | 26 breast cancer, 27 prostate cancer |  | ^18^F-NaF PET/CT | BS, SPECT, SPECT/CT, WB-MRI + DWI | Confirmed high risk breast and prostate cancer | NR | NR | Region based diagnostic performance (sensitivity, specificity, accuracy):  NaF PET/CT- 93%, 99%, 98%  WB-MRI + DWI- 91%, 99%, 97%  SPECT/CT- 85%, 99%, 96%  SPECT- 74%, 94%, 89%  BS- 62%, 98%, 90% |
| Janssen et.al [4] | Retrospective  (2017) | 54 | ^68^Ga-PSMA-11 | ^99m^-Tc-DPD-SPECT/CT |  | Prostate cancer patients referred for skeletal staging | NR | NR | Region based diagnostic performance (sensitivity, specificity, AUC):  PSMA PET/CT- 98%, 100%, 0.99  SPECT/CT- 69%, 98%, 0.84 |
| Lengana et.al [5] | Prospective  (2018) | 113 | ^68^Ga-PSMA-11 | BS |  | Confirmed prostate cancer patients underwent skeletal staging | 67 y | NR | Diagnostic performance in detecting skeletal lesions (sensitivity, specificity, PPV, NPV, accuracy):  PSMA PET/CT- 96%, 100%, 100%, 99%, 99%  BS- 73%, 87%, 63%, 92%, 84% |
| Poulsen et.al [6] | Prospective (2013) | 50 |  | ^18^F-NaF PET/CT | ^18^F-FCH PET/CT, BS | Confirmed prostate cancer patients consistent with bone metastases | 73 y | 84 | Diagnostic performance in detecting skeletal lesions (sensitivity, specificity, PPV, NPV, accuracy):  NaF PET/CT- 93%, 54%, 82%, 78%, 81%  FCH PET/CT- 85%, 91%, 95%, 75%, 87%  BS- 51%, 82%, 86%, 43%, 61% |
| Pyka et.al [7] | Retrospective (2016) | 126 | ^68^Ga-PSMA-11 | BS |  | Confirmed PCa patients referred for initial staging (n=37), and recurrence (n=89) | 69 y | 43.5 | Patient based diagnostic performance (sensitivity, specificity, AUC):  PSMA PET/CT – 100%, 100%, 0.99  BS- 89%, 96%, 0.90 |
| Zacho et.al [8] | Prospective (2018) | 68 | ^68^Ga-PSMA-11 | ^18^F-NaF PET/CT | DW-MRI | Confirmed PCa patients with biochemical recurrence | NR | NR | Patient based diagnostic performance (sensitivity, specificity, AUC):  PSMA PET/CT – 80%, 98%, 0.89  NaF PET/CT- 90%, 90%, 0.90  DW-MRI- 38%, 87%, 0.62 |
| Zacho et.al [9] | Retrospective (2020) | 112 | ^68^Ga-PSMA-11 |  | BS | Confirmed intermediate-to-high risk PCa at the primary staging | 68 y | 21 | Patient based diagnostic performance (sensitivity, specificity, PPV, NPV):  PSMA PET/CT – 100%, 93%, 74%, 100% |

BS = bone scintigraphy, CT = computed tomography, PET = positron emission tomography, SPECT = single photon emission tomography, WB-MRI = whole body magnetic resonance imaging, DWI = diffusion weighted imaging, PSMA = prostate specific membrane antigen, NaF = sodium fluoride, FCH = fluorocholine, ^99m^Tc-DPD = ^99metastable^ technetium-diphosphopropanedicarbonacid NR = not reported.

1. Dyrberg E, Hendel HW, Huynh THV, Klausen TW, Løgager VB, Madsen C et al. 68Ga-PSMA-PET/CT in comparison with 18F-fluoride-PET/CT and whole-body MRI for the detection of bone metastases in patients with prostate cancer: a prospective diagnostic accuracy study. European Radiology. 2019;29(3):1221-30.

2. Fonager RF, Zacho HD, Langkilde NC, Fledelius J, Ejlersen JA, Haarmark C et al. Diagnostic test accuracy study of (18)F-sodium fluoride PET/CT, (99m)Tc-labelled diphosphonate SPECT/CT, and planar bone scintigraphy for diagnosis of bone metastases in newly diagnosed, high-risk prostate cancer. American journal of nuclear medicine and molecular imaging. 2017;7(5):218-27.

3. Jambor I, Kuisma A, Ramadan S, Huovinen R, Sandell M, Kajander S et al. Prospective evaluation of planar bone scintigraphy, SPECT, SPECT/CT, 18F-NaF PET/CT and whole body 1.5T MRI, including DWI, for the detection of bone metastases in high risk breast and prostate cancer patients: SKELETA clinical trial. Acta Oncologica. 2016;55(1):59-67.

4. Janssen J-C, Meißner S, Woythal N, Prasad V, Brenner W, Diederichs G et al. Comparison of hybrid 68Ga-PSMA-PET/CT and 99mTc-DPD-SPECT/CT for the detection of bone metastases in prostate cancer patients: Additional value of morphologic information from low dose CT. European Radiology. 2018;28(2):610-9.

5. Lengana T, Lawal IO, Boshomane TG, Popoola GO, Mokoala KMG, Moshokoa E et al. 68Ga-PSMA PET/CT Replacing Bone Scan in the Initial Staging of Skeletal Metastasis in Prostate Cancer: A Fait Accompli? Clinical Genitourinary Cancer. 2018;16(5):392-401.

6. Poulsen MH, Petersen H, Høilund-Carlsen PF, Jakobsen JS, Gerke O, Karstoft J et al. Spine metastases in prostate cancer: comparison of technetium-99m-MDP whole-body bone scintigraphy, [18F]choline positron emission tomography(PET)/computed tomography (CT) and [18F]NaF PET/CT. BJU International. 2014;114(6):818-23.

7. Pyka T, Okamoto S, Dahlbender M, Tauber R, Retz M, Heck M et al. Comparison of bone scintigraphy and 68Ga-PSMA PET for skeletal staging in prostate cancer. European Journal of Nuclear Medicine and Molecular Imaging. 2016;43(12):2114-21.

8. Zacho HD, Nielsen JB, Afshar-Oromieh A, Haberkorn U, deSouza N, De Paepe K et al. Prospective comparison of 68Ga-PSMA PET/CT, 18F-sodium fluoride PET/CT and diffusion weighted-MRI at for the detection of bone metastases in biochemically recurrent prostate cancer. European Journal of Nuclear Medicine and Molecular Imaging. 2018;45(11):1884-97.

9. Zacho HD, Ravn S, Afshar-Oromieh A, Fledelius J, Ejlersen JA, Petersen LJ. Added value of (68)Ga-PSMA PET/CT for the detection of bone metastases in patients with newly diagnosed prostate cancer and a previous (99m)Tc bone scintigraphy. EJNMMI research. 2020;10(1):31-.
